# DISH as a marker for incident diabetes mellitus in cardiovascular disease patients

**DOI:** 10.1093/rheumatology/keaf268

**Published:** 2025-05-27

**Authors:** Netanja I Harlianto, Wouter Foppen, Firdaus A A Mohamed Hoesein, Marjolein E Hol, Jorrit-Jan Verlaan, Pim A de Jong, Jan Westerink, UCC-SMART Study group, M J Cramer, M G van der Meer, P van der Harst, H M Nathoe, J van Setten, M Teraa, N C Onland-Moret, M van Smeden, M H Emmelot-Vonk, P A de Jong, A T Lely, S Haitjema, M M Mokhles, Y M Ruigrok, M C Verhaar, J A N Dorresteijn, F L J Visseren

**Affiliations:** Department of Radiology, University Medical Center Utrecht and Utrecht University, Utrecht, The Netherlands; Department of Orthopedic Surgery, University Medical Center Utrecht and Utrecht University, Utrecht, The Netherlands; Department of Radiation Oncology, University Medical Center Utrecht and Utrecht University, Utrecht, The Netherlands; Department of Radiology, University Medical Center Utrecht and Utrecht University, Utrecht, The Netherlands; Department of Radiology, University Medical Center Utrecht and Utrecht University, Utrecht, The Netherlands; Department of Radiology, University Medical Center Utrecht and Utrecht University, Utrecht, The Netherlands; Department of Orthopedic Surgery, University Medical Center Utrecht and Utrecht University, Utrecht, The Netherlands; Department of Radiation Oncology, University Medical Center Utrecht and Utrecht University, Utrecht, The Netherlands; Department of Radiology, University Medical Center Utrecht and Utrecht University, Utrecht, The Netherlands; Department of Internal Medicine, Isala Zwolle, Zwolle, The Netherlands

**Keywords:** DISH, diabetes mellitus, incidence rate: prospective cohort

## Abstract

**Objectives:**

DISH is a common incidental finding in medical imaging characterized by continuous vertebral ossification, which is associated with prevalent type 2 diabetes mellitus (T2DM). We hypothesized that incidental screen-detected DISH may be an actionable marker for incident diabetes screening and aimed to assess the absolute incidence rate (ratio) for T2DM in cardiovascular patients with and without DISH.

**Methods:**

Cardiovascular disease patients without diabetes (*n* = 3395) were included via the prospective Second Manifestation of ARTerial disease cohort. DISH was evaluated at baseline on chest radiographs using Resnick criteria. Incident T2DM was assessed by an adjudication committee. Adjusted incidence rate ratios (IRRs) and numbers needed to screen were calculated.

**Results:**

DISH was present in 263 (7.7%) patients. After a median follow-up of 11.1 years (IQR: 7.1–15.2), 317 patients developed T2DM. Patients with DISH had a higher incidence rate for T2DM compared with no-DISH patients (17.1 vs. 7.8 T2DM per 1000 person-years). DISH was associated with incident T2DM in multivariate analyses (IRR: 1.47; 95% CI: 1.03–2.06), with the highest IRR in the DISH group with the most extensive ossification (IRR: 2.01; 95% CI: 1.15–3.29). The number needed to screen for T2DM in patients with screen-detected DISH for 11.1 years was 7, similar to accepted risk markers overweight (*n* = 8), obesity (*n* = 5), hypertension (*n* = 9) and hyperlipidaemia (*n* = 13).

**Conclusions:**

DISH is associated with higher rates of incident T2DM in cardiovascular disease patients, independent of accepted risk markers. DISH could be used as an imaging marker to identify cardiovascular disease patients with an increased risk for subsequent T2DM.

## Graphical Abstract

**Figure:**
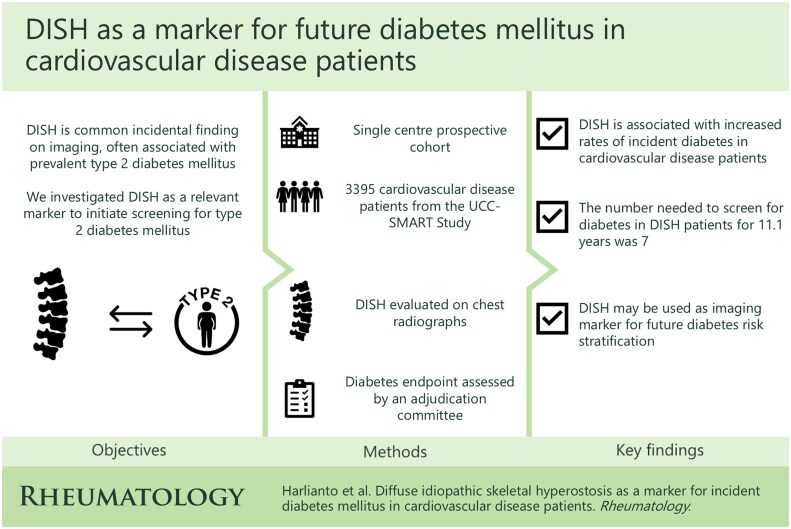

Rheumatology key messages

- DISH is associated with increased rates of incident diabetes in patients with cardiovascular disease.
- DISH patients with >7 fused vertebral bodies had a 2-fold higher risk for incident diabetes.
- DISH may be used as imaging marker for future diabetes risk stratification.

## Introduction

**Figure 1.**
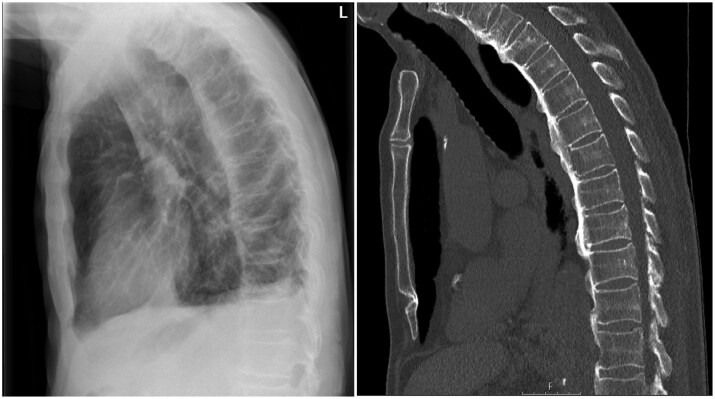
Grade III DISH on a lateral chest radiograph (left) and sagittal computed tomography scan (right) in a 55-year-old patient. Note the bridging ossification, which cannot be explained by disc degeneration. Also note the absence of facet joint ankylosis which differentiates DISH from ankylosis spondylitis

DISH is a systemic disorder characterized by the progressive formation of osseous bridging near the anterolateral spine [1]. DISH was first described in 1950 by Forestier and Rotes-Querol, and is also known as Forestier's disease [2]. Diagnostic criteria for DISH have been described by Resnick and Niwayama in 1976 [3], which to date, have become the most commonly used criteria. DISH is diagnosed when the following criteria are met: (1) bridging ossification anterior to the spine with involvement of at least three contiguous intervertebral bridges of ossification; (2) with preservation of the intervertebral disc space to differentiate DISH from degenerative disc disease and (3) the absence of ankylosis of the sacroiliac or facet joints to differentiate DISH from spondyloarthritis. An example of DISH on radiographs and computed tomography is shown in Fig. 1. DISH is commonly seen in males over the age of 50 years, with a prevalence of over 10% of the population, which is increasing with age [1, 4]. Although largely asymptomatic, patients with DISH may experience stiffness and back pain [4, 5]. Moreover, at the level of the cervical spine, this ossification may result in symptoms of airway compression and dysphagia [6]. The exact pathophysiology remains to be established, but DISH is associated with obesity, hypertension, metabolic syndrome [7], vascular calcifications [8–10] and ischaemic stroke [11]. Several cross-sectional studies observed an association with type 2 diabetes mellitus (T2DM) [4, 11, 12]. DISH and T2DM share characteristics such as metabolic disturbances with insulin resistance, adipose tissue dysfunction and low-grade inflammation [13–15]. Whether DISH is a possible clinically relevant marker for incident diabetes mellitus in daily practice remains unknown, as this association has not been studied longitudinally [16].

DISH is a common incidental imaging finding, e.g. on chest radiography, the most commonly ordered radiological test, with an annual number of 129M in the USA [17]. Additionally, the number of chest CT scans is increasing in clinical medicine, as well as in lung cancer and cardiovascular screening. Hence, DISH status can be made available for a substantial number of the population on examinations that are acquired for other purposes. The American Diabetes Association Guidelines recommend screening for diabetes in (asymptomatic) subjects with an increased cardiovascular disease burden, including obesity, hypertension, decreased high density lipoprotein cholesterol (HDL) [18]. We hypothesized that DISH could be a marker with similar strength and thus be a relevant incidental imaging finding to initiate screening for T2DM. Thus, the aim of the current study was to evaluate the risk for incident T2DM in patients with DISH in a prospective cohort study of cardiovascular disease patients.

## Methods

### Study cohort

Our patient cohort is derived from the Second Manifestations of ARTerial disease (UCC-SMART) study, an ongoing prospective cohort study initiated in 1996, which follows patients between the ages of 18 and 79 years with either manifest or risk factors for vascular disease [19]. The UCC-SMART study was approved by the medical ethics committee of the UMC Utrecht, adhered to the Declaration of Helsinki, and all included patients gave written informed consent. The current study included all patients who underwent a digital chest radiograph for various clinical indications outside participation in the UCC-SMART cohort within three months of inclusion. We excluded patients where DISH could not be adequately assessed on imaging, patients with diabetes mellitus at baseline and subjects without a history of diabetes with a serum glucose at baseline higher than 7 mmol/l or Hba1c at baseline higher than 6.5%. Diabetes mellitus at baseline was defined as either a referral diagnosis of diabetes, self-reported diabetes including use of glucose-lowering agents, glucose ≥11.1 mmol/l or initiation of glucose-lowering treatment within one year after inclusion with a glucose ≥7.0 mmol/l at baseline. The reporting of the current study adhered to the Strengthening the Reporting of Observational Studies in Epidemiology (STROBE) guidelines [20].

### Physical and laboratory examinations

Extensive vascular screening was performed for all included patients. This consisted of a health questionnaire on medical history, risk factors, smoking and drinking habits, and prescribed drugs. According to a standard diagnostic protocol, physical examination and laboratory testing in a fasting state were also performed [19]. BMI was calculated as weight divided by height squared (kg/m^2^). Blood pressure was measured automatically, in triplicate with a 30-s interval, with the mean value of three measurements was used. Hypertension was defined as systolic blood pressure ≥140 mmHg and/or diastolic blood pressure ≥90 mmHg and/or use of antihypertensive medication. In a fasting state, blood samples were collected for HbA1c, glucose, blood lipids, high sensitive CRP (hsCRP) and creatinine levels. Renal function was estimated by means of the Chronic Kidney Disease Epidemiology Collaboration equation (CKDEPI) [21]. Hyperlipidaemia was defined as low-density lipoprotein (LDL)-cholesterol ≥2.6 mmol/l [22].

### DISH presence and extent

Using the Resnick criteria [3], DISH was evaluated on anteroposterior chest radiographs by a group of six independent readers from the department of Radiology of our institution. Reflecting clinical practice the chest radiographs were interpreted by one of the six readers all of whom were certified to independently read chest radiographs, as described previously [11]. The readers were blinded to incident T2DM status. DISH extent was scored as the following: grade 1 DISH: ossification of four adjacent vertebral bodies; grade 2 DISH: ossification of five or six adjacent vertebral bodies; grade 3 DISH: ossification of seven or more adjacent vertebral bodies.

### Follow-up and diabetes end point assessment

The primary end point for our study was incident T2DM [19]. All patients without diabetes at baseline included until June 2006 received a questionnaire between June and December 2006 to evaluate T2DM incidence following study inclusion. From 2006 onwards, patients were asked to complete biannual questionnaires whether they had diabetes. In the case of ‘yes’, patients received an additional questionnaire regarding the date of diagnosis, initial treatment (diet, oral medication or insulin), current treatment and family history of diabetes. Patients or their general practitioner were contacted by telephone for additional information, in the case of incomplete or unclear answers. Diabetes events were audited and classified by two independent physicians, in order to validate the diagnosis. Cross-validation with the hospital diagnosis registry showed that none of the patients who reported not to have diabetes mellitus had a physician’s diagnosis of diabetes. Follow-up was defined as the time between study inclusion and the date of diagnosis of T2DM, death from any cause, loss to follow-up or the preselected date of January 2023.

### Statistical analysis

Normal distributed data were expressed using the mean and standard deviation, and categorical variables using frequencies and percentages. Incidence rates were calculated for the DISH and no-DISH groups. Moreover, the effect of DISH on the incidence rate ratio (IRR) of incident T2DM occurrence with corresponding 95% confidence intervals (95% CIs) was estimated using Poisson regression models, for which the event count was the dependent variable and the individual follow-up time was included in the model as an offset. Continuous variables were transformed if they were not linearly related to the number of events. We accounted for over- or under-dispersion (i.e. less or more spread in the individual count observations than expected on the basis of the Poisson distribution). Adjustments were made for age and sex, and additionally for BMI, systolic blood pressure, non-HDL cholesterol and CKDEPI. Finally, we calculated the number needed to screen as one divided by the crude incidence rate for the overall DISH groups, and groups stratified by overweight and DISH subjects. Missing covariate data were imputed using single regression imputation for non-HDL (*n* = 12, 0.4%), CKDEPI (*n* = 9, 0.3%), systolic blood pressure (*n* = 4, 0.1%) and BMI (*n* = 5, 0.1%). Statistical significance was deemed at *P*-value <0.05. Statistical analysis was performed with R, version 4.3.3 (R Foundation for Statistical Computing, Vienna, Austria).

### Patient and public involvement

Patients and/or the public were not involved in the design, or conduct, or reporting, or dissemination plans of this research.

## Results

### Patient characteristics

**Figure 2.**
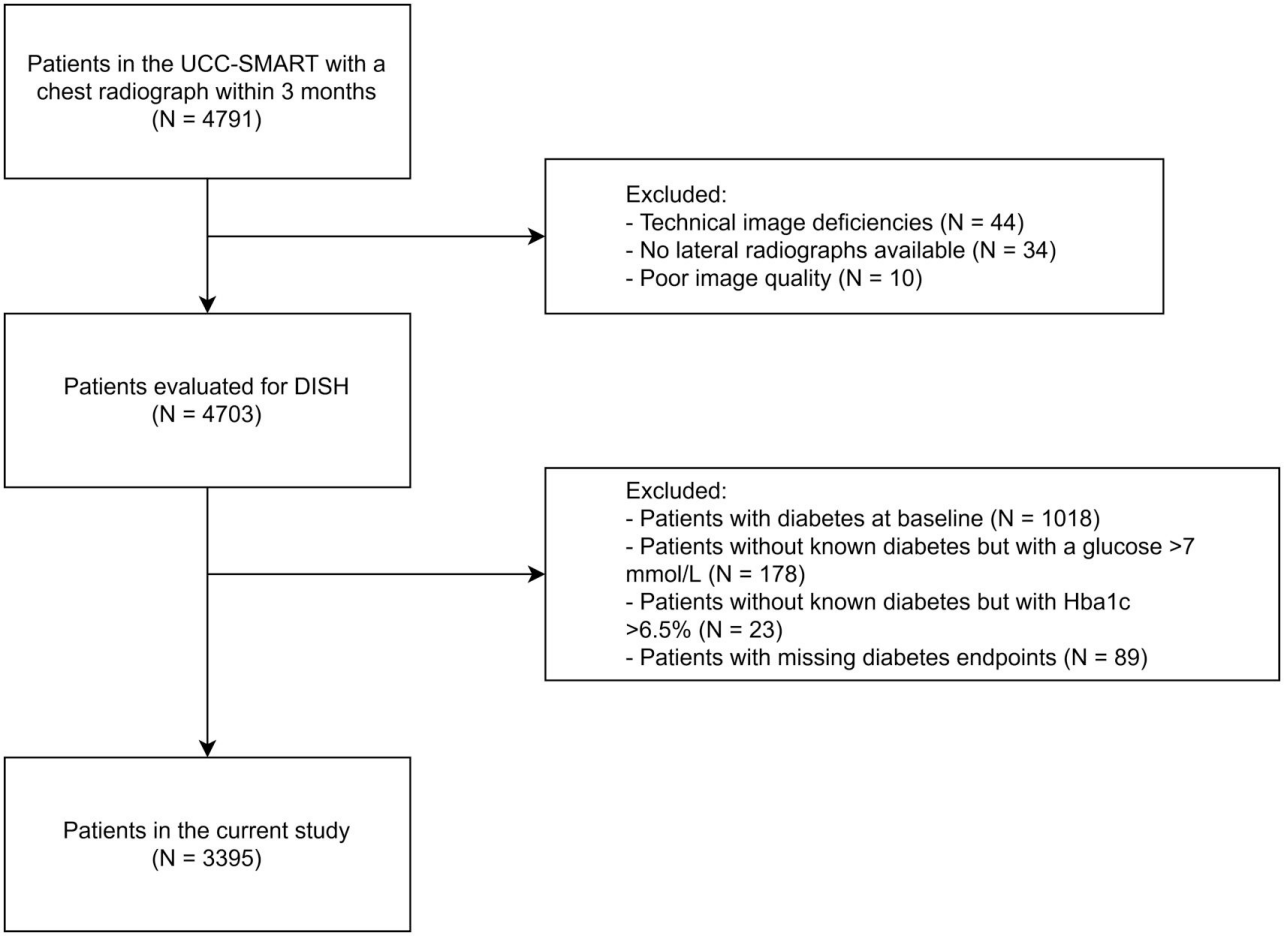
Flowchart of patient selection

We identified 4791 patients, of which 88 were excluded due to technical image deficiencies (*n* = 44), availability of only anteroposterior radiograph (*n* = 34) and poor image quality (*n* = 10). Following this, we excluded all patients with diabetes mellitus at baseline (*n* = 1018), without a known history of diabetes but with a glucose at baseline higher than 7 mmol/l (*n* = 178) or with Hba1c at baseline higher than 6.5% (*n* = 23), and with missing diabetes end point (*n* = 89). Ultimately, 3395 patients were eligible for the current study (Fig. 2).

DISH was present in 263 patients (7.7%), including 99 patients with grade 1 DISH, 82 patients with grade 2 DISH and 82 patients with grade 3 DISH. A total of 317 patients (9.3%) developed T2DM during the follow-up period (median follow-up of 11.1 years; interquartile range: 7.1–15.2 years). A full overview is provided in Table 1.

**Table 1.** Baseline table and factors associated with incident diabetes mellitus

Variable	No incident diabetes (*n* = 3078)	Incident diabetes (*n* = 317)
DISH	7.3%	12.3%
Age in years	57.9 (11.4)	56.2 (9.8)
Male sex	68.5%	76.0%
BMI, kg/m^2^	26.2 (4.0)	29.0 (4.3)
Overweight	60%	73%
Obesity	35%	48%
Glucose, mmol/l	5.7 (0.6)	6.4 (0.9)
HbA1c in %	5.5 (0.4)	5.9 (0.5)
Systolic blood pressure, mmHg	140 (22)	142 (23)
Hypertension	24.0%	28.7%
Non-HDL, mmol/l	3.6 (1.2)	4.0 (1.5)
Hyperlipidaemia	43.8%	50.5%
CRP, mg/l	3.9 (7.7)	3.9 (5.7)
CKDEPI, ml/min/1.73 m^2^	79 (18)	78 (18)
Former or current smoker	72.6%	78.1%
Packyears	16.5 (18.9)	20.9 (20.6)
History of vascular disease	72.5%	73.1%
Statin use	58.9%	67.7%
Ezitimibe use	5.1%	4.8%

Numerical data expressed as mean (standard deviation), categorical data as absolute (percentage). HDL: high density lipoprotein; CKDEPI: chronic kidney disease Epidemiology Collaboration.

### IRRs for incident T2DM

Incidence rates for the total cohort, DISH group and no-DISH group are shown in Table 2. Subjects without DISH had an incidence rate of 7.8 per 1000 person-years for T2DM, which was higher for patients with DISH at 17.1 per 1000 person-years. Stratified by DISH severity, incidence rates increased with increasing severity: 11.3 per 1000 person-years for grade 1 DISH; 12.5 per 1000 person-years for grade 2 DISH and 19.2 per 1000 person-years for grade 3 DISH.

**Table 2.** Incidence rates for incident diabetes mellitus

	Total group (*N* = 3395)	No-DISH (*N* = 3132)	Any DISH (*N* = 263)	Grade 1 DISH (*N* = 99)	Grade 2 DISH (*N* = 82)	Grade 3 DISH (*N* = 82)
Sum person-years	38 290	35 520	2770	1060	877	833
No. of diabetes cases	317	278	39	12	11	16
Incidence rate per 1000 person-years	8.3	7.8	17.1	11.3	12.5	19.2

Poisson regression adjusted RR is provided in Table 3. For any DISH, the crude RR for incident T2DM was 1.80 (95% CI: 1.27–2.48). After adjusting for other factors that may lead to a higher rate of incident T2DM, patients with DISH had a 1.47 (95% CI: 1.03–2.06) higher incidence rate of T2DM during the follow-up period, compared with patients without DISH. These increases were most pronounced in the group with the most severe ossification (grade 3 DISH), with a 2.01 (95% CI: 1.15–3.29) higher incidence rate of T2DM compared with subjects without DISH. In addition, incidence rates were higher for subjects that were overweight (RR: 3.01; 95% CI: 2.26–4.10) or obese (RR: 2.99; 95% CI: 2.36–3.77), whereas this was not observed for the presence of hypertension or hyperlipidaemia.

**Table 3.** Incidence rate ratios and number needed to screen for incident diabetes

	Any DISH	Grade 1 DISH	Grade 2 DISH	Grade 3 DISH	BMI > 25 kg/m^2^	BMI > 30 kg/m^2^	Hypertension	Hyperlipidaemia
Crude IRR	1.80 (1.27–2.48)	1.45 (0.76–2.46)	1.60 (0.82–2.78)	2.45 (1.42–3.92)	3.15 (2.37–4.27)	2.96 (2.34–3.71)	1.22 (0.96–1.56)	1.16 (0.92-1.45)
Age, sex adjusted IRR	1.84 (1.28–2.57)	1.51 (0.79–2.58)	1.59 (0.81–2.79)	2.55 (1.46–4.15)	3.12 (2.35–4.23)	3.00 (2.37–3.77)	1.27 (0.99–1.62)	1.19 (0.95-1.50)
Age, sex, BMI adjusted IRR	1.50 (1.04–2.10)	1.21 (0.64–2.07)	1.27 (0.65–2.22)	2.20 (1.26–3.58)	–	–	1.11 (0.86–1.41)	1.25 (0.99-1.58)
Full adjusted IRR	1.47 (1.03–2.06)	1.19 (0.63–2.04)	1.31 (0.67–2.29)	2.01 (1.15–3.29)	3.01 (2.26–4.10)	2.99 (2.36–3.77)	1.06 (0.82–1.36)	1.24 (0.98-1.56)
Number needed to screen for 11.1 years	7	8	7	5	8	5	9	13

Adjusted IRR with 95% confidence intervals are calculated with poison regression. Fully adjusted model adjusted for age, sex, BMI, systolic blood pressure, log-transformed non-HDL, CKDEPI. Number needed to screen is one divided by the observed incidence over the follow-up period. IRR: incidence rate ratio.

We calculated that the follow-up of seven DISH patients with cardiovascular disease for 11.1 years would detect one case of T2DM during our study follow-up period (T2DM incidence in DISH: 14.8%). For overweight subjects, follow-up of eight patients would detect one case of T2DM (T2DM incidence 12.6%), which was five patients per case for obesity (T2DM incidence 19.7%), 13 patients per case for hyperlipidaemia (T2DM incidence 7.7%) and nine patients per case for hypertension (T2DM incidence 11.0%). In overweight subjects with DISH (*n* = 193), the incidence of T2DM was 16.1% (six cases of follow-up); which was 12.3% for overweight subjects without DISH (eight cases). In contrast, in subjects with a BMI < 25 kg/m^2^ without DISH (*n* = 1245), this was 3.7% (27 cases of follow-up), whereas 11.4% of patients with DISH and a BMI < 25 kg/m^2^ had incident T2DM (nine cases of follow-up).

## Discussion

The current study aimed to evaluate the absolute risk of T2DM development in patients with DISH in a cohort of cardiovascular disease patients in comparison with established screening indicators for diabetes. We quantified the total event rate for T2DM and found that the presence of DISH was associated with an increased rate of incident T2DM. This was most pronounced for subjects with extensive vertebral ossification (grade 3 DISH: >7 fused vertebral bodies), which had a 2-fold higher risk for incident T2DM compared with subjects without DISH. Moreover, this observed association was fairly similar to obesity and overweight subjects and stronger than hypertension and hyperlipidaemia. In our cohort seven DISH patients would need to be screened for 11 years to identify one T2DM potentially earlier. Our study highlights the potential role of incidentally discovered DISH on available medical images as a marker to stratify cardiovascular disease patients with an increased risk of developing incident T2DM.

DISH can be easily evaluated on various radiological examinations. Worldwide, the demand for radiological examinations continues to rise [18, 23], as well as a rise in the number of T2DM [24] and DISH cases. A broad range of imaging can be used to assess DISH where the spine is visualized. These include chest radiographs, spinal radiographs, cervico-thoraco-abdominal CT and MRI images, as well as Dual-Energy X-ray absorptiometry (DXA) and dental radiological examinations [16, 25]. Patients with T2DM have significant risks for various cardiovascular complications, constituting a major health problem worldwide [24]. Similarly, it is estimated that T2DM is underdiagnosed in a large portion of the population [26]. It is established that early identification of T2DM is relevant, and guidelines propose several routes for screening high risk groups. The European Society of Cardiology recommends diabetes screening in all individuals with cardiovascular disease by means of fasting glucose or Hba1c [27]. The American Diabetes Association recommends screening in obese patients, hypertensive patients or subjects with dysregulated lipids. In our cohort, this would translate in number needed to screen for 11.1 years of five obese patients to detect one case of T2DM, which was nine cases for hypertension and 13 cases for hyperlipidaemia. Within these European guidelines, no specific screening interval is mentioned [27], whereas the American guideline recommends screening of high risk individuals within 3 years or less [18]. We found that the follow-up of seven patients with DISH would detect one case of T2DM in our study follow-up period, which was as low as five patients to detect one case of T2DM for the most severe DISH group. One could argue that these patients are all overweight and that DISH has no added value as a risk marker. However, notably, in subjects with a BMI lower than 25 kg/m^2^, a higher percentage of incident T2DM was found in the DISH group compared with patients without DISH (11.4% vs 3.7%), whereas this difference was less pronounced for overweight subjects (16.1% vs 12.3%). The multivariable poison regression also showed that overall, the presence of DISH was associated with a higher rate of T2DM, irrespective of BMI. We therefore provide evidence that in the case DISH is incidentally found on imaging in a patient without known diabetes mellitus, repeated laboratory testing may be recommended to assess whether diabetes is present or develops over time. We propose external validation and possibly (cost-)effectiveness evaluation, although the diagnosis of DISH is a free add-on on medical imaging and costs of screening are low.

Although for diagnosis and screening pathophysiological knowledge is not essential, previous cross-sectional studies have shown that DISH subjects have an increased prevalence of diabetes mellitus in both cardiovascular disease cohorts, as well as samples derived from the general population [4, 11, 12]. It remains uncertain whether DISH may be a result of insulin resistance and diabetes, or whether diabetes leads to anterolateral bone deposition and the development of DISH. It has previously been suggested that hypertension and diabetes are potential risk factors for early DISH. This was based on a small case and control study of 62 subjects, matched for age and sex [28]. Patients with DISH have demonstrated increased levels of growth hormone and insulin. It has been previously hypothesized that growth factors for bone formation may be related to metabolic imbalances in the insulin-like growth factor-I pathway, which is responsible for osteoblast and chondrocyte proliferation [29]. In contrast, another study found that DISH patients have impaired beta-cell pancreatic stimulation, as well as higher insulin hepatic extraction. They suggested that this dysregulation could result in abnormal insulin sensitivity and insulin resistance [14]. Two previous studies have demonstrated that DISH patients have lower levels of adiponectin [30, 31], an adipokine that is associated with an increased risk in a dose response relationship for incident T2DM [32]. In these cohorts, patients with DISH also had higher levels of visfatin [30] and leptin [31]. These studies may partially explain the relation between DISH and incident T2DM. Increasing interest is being given to a possible inflammatory component in DISH, in addition to metabolic factors. This is supported by imaging studies with some similarities between DISH and other inflammatory rheumatic diseases [33]. DISH presence has also been found to affect anti-inflammatory treatment outcomes in other rheumatic disorders [34]. In our cohort, we had no extensive information available on whether patients suffered from rheumatic diseases and used anti-inflammatory therapy, which would have been of interest to explore in relation to DISH and T2DM development. We had information on inflammation using laboratory high-sensitivity CRP, which did not differ between patients that did and did not develop T2DM. The exact pathophysiology remains complex. We believe that it would be of interest for future work to evaluate the progression and development of DISH in a population of T2DM patients, to learn more about the pathophysiology.

Our study has multiple strengths. First, we used data from a large prospective study with uniform and standardized data collection at baseline. Second, we were able to assess associations with adjustments due to extensive data collection of cardiovascular determinants. Finally, our study population had long and complete follow-up with end point adjudication by multiple physicians, which reduced end point subjectivity assessment.

The limitations of our study should also be considered. The selection of patients with chest radiographs could have resulted in the selection of subjects with a recent cardiovascular event, which could have resulted in increased selection of subjects from the UCC-SMART cohort with a higher risk. Independent of this, the UCC-SMART cohort is not a reflection of a general population or patients presenting at a general practitioner. As our study population of patients with known cardiovascular disease is enriched with known risk factors for DISH, the numbers needed to screen are presumably higher than in a lower risk population without cardiovascular disease. Therefore, we recommend external validation of our findings in a general population or first/second line medicine cohort. Another limitation is that DISH presence was not assessed at multiple time-points or at the time of T2DM event occurrence. We believe that this would have resulted in stronger associations with DISH. Therefore, future studies could also incorporate both DISH and T2DM assessment across multiple time-points. Third, we had no data available on anti-inflammatory rheumatic drugs usage in our population. However, our results were not affected by statin and anti-cholesterol therapy usage, medication characterized by its anti-inflammatory properties [35]. Finally, we have no data available on the interrater reliability of DISH, but our readings reflect clinical practice, where a single reader provides the diagnosis to the referring physician and the patient. Chest radiographs are a valid tool for DISH diagnosis, with high interrater reliability (Cohen’s kappa: 0.93) [36].

## Conclusion

The presence of DISH is associated with increased rates of incident T2DM in patients with cardiovascular disease at a level similar to accepted screening criteria for incident T2DM. We found that this association was most pronounced in the DISH group with the most extensive ossification, with a 2-fold higher risk for T2DM compared with subjects without DISH. Depending on replication, DISH could be used as an imaging marker to identify individuals who have an increased risk for subsequent T2DM development.

## Data Availability

The informed consent that was signed by the study participants is not compliant with publishing individual data in an open access institutional repository or as supporting information files with the published article. However, a data request can be sent to the SMART Steering Committee at uccdatarequest@umcutrecht.nl.
